# Clinical Commentary: Obstetric and Respiratory Management of Pregnancy with Severe Spinal Muscular Atrophy

**DOI:** 10.1155/2009/942301

**Published:** 2009-05-19

**Authors:** Daniel Flunt, Natasha Andreadis, Collette Menadue, Alec W. Welsh

**Affiliations:** ^1^Department of Respiratory and Sleep Medicine, The Royal Prince Alfred Hospital, Missenden Road, Camperdown, NSW 2050, Australia; ^2^Department of Obstetrics and Gynaecology, The Royal Prince Alfred Hospital, Missenden Road, Camperdown, NSW 2050, Australia; ^3^Department of Maternal-Fetal Medicine, The Royal Hospital for Women, University of New South Wales, Barker Street, Randwick, NSW 2031, Australia

## Abstract

We present a combined obstetric and respiratory perspective on two pregnancies for a woman with severe Type 2 Spinal Muscular Atrophy (SMA). Our patient had the lowest prepregnancy weight (20 kg) and vital capacity of 0.34 L (VC 11% predicted) yet to be reported in the sparse literature on pregnancy with SMA. She delivered two live healthy infants via planned caesarean section without pregnancy or neonatal complication. We describe the respiratory and obstetric management techniques used for a pregnancy with this degree of respiratory compromise.

## 1. Introduction

Spinal Muscular Atrophy (SMA) has an incidence of 1/6000–1/10000 and a carrier frequency of 1/40 to 1/50 [1], being the second commonest autosomal recessive disorder in whites after cystic fibrosis [2]. It is characterized by degeneration of alpha neurons in the anterior horn cells of the spinal cord leading to progressive muscle atrophy and premature death, usually from respiratory failure. Type 1 SMA (the most severe) presents with weakness at birth or in the first six months of life. Type 2 SMA presents between six and 18 months of age with weakness of muscles in the legs and trunk and failure to meet motor milestones (crawling and walking). Prognosis is dependent on the extent and timing of respiratory complications. Inspiratory muscle weakness predisposes the individual to ventilatory failure, and expiratory muscle impairment causes an ineffective cough, which can lead to secretion retention and chronic atelectasis [3]. Type 3 SMA presents after 18 months of age with the ability to walk which may be lost in time. 

We report two successful pregnancies in a woman with Type 2 SMA, with a vital capacity (VC) of 0.34 L (11% of predicted value [4]) and baseline inspiratory and expiratory muscle strengths of −27 cmH_2_O (41% predicted) and +19 cmH_2_O (22% predicted), respectively. Successful pregnancies in patients with Type 2 SMA have been reported previously but at baseline vital capacities of 0.6 to 1.98 L [5, 6] and percent predicted values of 30 to 67% [7–9]. Pregnancy has also been reported in a tracheostomised patient with Type 1 SMA (VC 0.47 L) [10] and in three postpoliomyelitis patients, continuously dependent on noninvasive ventilation (NIV), with vital capacities as low as 0.24 L to 0.28 L [11]. Our case highlights the importance of multidisciplinary antenatal care in managing such a condition during pregnancy and in achieving the optimal outcome for both mother and baby.

## 2. Case Report

A 32-year-old woman presented to a high-risk pregnancy clinic in her first ongoing pregnancy. Developmentally, her milestones had been classically delayed, crawling at age one. She slowly developed severe contractures of her limbs and severe kyphoscoliosis throughout her teenage years. At age 17, she presented with compensated hypercapnic respiratory failure (pH 7.41, PaCO_2_ 57 mmHg, PaO_2_ 48 mmHg, bicarbonate 36 mmol/L) and was commenced on nocturnal nasal assist-control volume ventilation for ventilatory insufficiency. Her daytime arterial blood gases (ABGs) normalized and stabilized with continued nocturnal NIV usage. She was above average in performance at school and completed a university degree. Diagnosis of Type 2 SMA with a homozygous deletion of the SMN 1 gene had been made at age 23. 

### 2.1. 1st Pregnancy

Antenatal booking weight was 20 kilograms, and the patient was wheelchair dependent and kept upright by means of a neck brace, with movement only of her left wrist and left fingers. Clexane thromboprophylaxis was commenced given her immobility (10 mg daily). The couple declined invasive genetic testing for SMA. First trimester screening gave a risk of approximately 1 : 2000 for Trisomy 21.

Outpatient respiratory monitoring was performed every second week from week 10 to 22, then weekly from week 22 to 26, including VC and peak cough flow rates. Sequential inspiratory and expiratory muscle strength measurement were attempted, but the patient did not have significant effort to register a measurement consistently. Baseline (10 weeks) ABGs were attempted, but abandoned due to the difficulty in obtaining a sample secondary to severe contractures in her wrists, elbows, and hips. For this reason, routine serial ABGs were not performed and due to a lack of equipment, end-tidal CO_2_ measures were not obtained. Room air oximetry served as a guide to gas exchange during her pregnancy, and ABGs measurements would have been performed in conjunction with deterioration in respiratory symptoms. 

Using the patient's own volume ventilator (VS Ultra, SAIME, Savigny le Temple, France; tidal volume 0.35 L, rate 22, inspiratory time 1.1 s), a mouthpiece (Hudson RCI, Temecula, Calif, USA) was introduced and she was shown how to “breath stack” thrice daily, to achieve maximal insufflation capacity and as a convenient method to supplement tidal breathing when awake. Training on the mechanical inexsufflator (CoughAssist, Respironics, Murrysville, Pa, USA) and manual assisted coughing was regularly practised as a precaution for secretion retention, with particular emphasis being placed on the importance of these techniques for the postoperative period. A variety of mask interfaces (including mouthpiece) were trialled to minimise potential pressure trauma to the nose and face from her usual nasal mask (Non-vented Sullivan-Bubble-Mask, ResMed, Sydney, Australia), in case she progressed to diurnal ventilator dependence. Significant respiratory compromise was anticipated. However, the timing and rate of respiratory deterioration were unable to be determined as domiciliary monitoring equipment such as a pulse oximeter, and devices such as a mechanical inexsufflator were not provided by the patient's health care system. 

**Figure 1:**
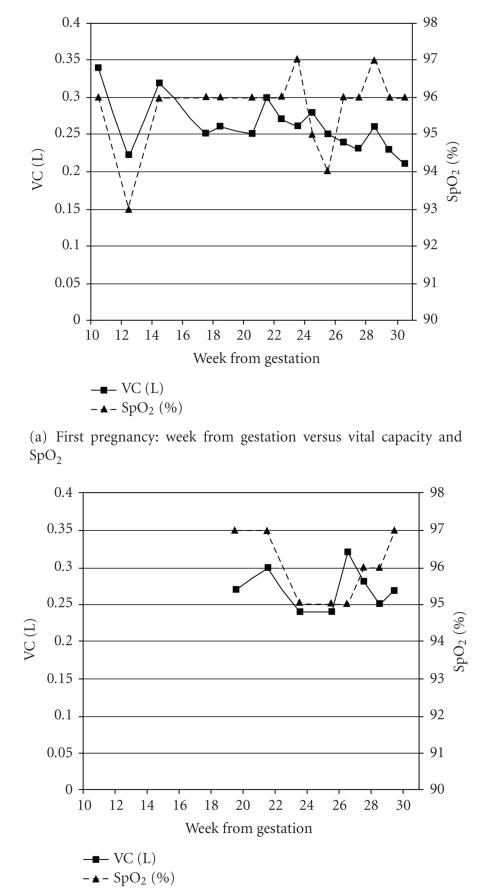
Vital capacity (VC) (solid line) and room air oxygen saturations (SpO_2_) (dashed line) measured at week from gestation for (a) the first and (b) the second pregnancies.

Respiratory function remained stable during the pregnancy, with VC ranging between 0.22 to 0.30 L and SpO_2_ on room air from 93 to 97%, as illustrated in Figure 1(a). Peak cough flow rates during the pregnancy ranged between 0.74 and 1.2 L/s. Ultrasonography at 24 weeks, 5 days showed an appropriately grown fetus weighing 0.629 kg (60th centile) with no evidence of compromise. 

Planned admission took place at 26 weeks gestation for daily respiratory observation and preparation for delivery by planned caesarean section at 28 weeks. The indication for mode of delivery was a combination of prematurity, kyphoscoliosis, anticipated cephalopelvic disproportion, and primiparity. The gestation was chosen as the best balance of fetal respiratory maturity and maternal respiratory function, anthropometry, home equipment constraints, and patient's preference for level of NIV dependency. Respiratory status was stable with SpO_2_ 96% on room air, respiratory rate (RR) of 26 breaths/min and VC 0.26 L. Antenatal admission allowed multidisciplinary review by anaesthetists, intensivists, neonatologists, midwives, physiotherapists, dieticians, occupational therapists, lactation consultants, and social workers. Respiratory function remained stable with the use of nocturnal NIV, with only transient complaints of dyspnoea on repositioning relieved by short bouts of day-time nasal ventilation. Preoperative VC measured 0.26 L and weight was 23.5 kg (BMI 9.6 kg/m^2^). Two doses of 11.4 mg betamethasone acetate were administered one week prior to delivery for fetal lung maturation.

At caesarean section, epidural space cannulation was successful yet the sensory block was unsatisfactory for the level of surgery, potentially due to a partial occlusion of the epidural space related to her severe kyphoscoliosis. Consequently, a general anaesthetic was administered with the assistance of mouthpiece volume ventilation to assist breathing during the awake fibre-optic nasal intubation. During the process of the intubation, supplemental oxygen was not required to be entrained through the mouthpiece ventilation, as transient falls in saturations were caused by suboptimal ventilation which occurred when the mouth piece seal was lost during moments of anxiety. This was instead corrected by manually assisting with the patient's mouthpiece seal, promoting calm in the patient and by increasing the volume being delivered by the ventilator (from 0.35 to 0.45 L) to cope with transient leaks. A midline abdominal incision was performed for access due to the kyphoscoliosis, with minimal subcutaneous fat and an atrophied rectus abdominis muscle being noted. An uneventful lower segment caesarean section was performed with delivery of a live female infant (Apgars of 7 at 1 minute, 9 at 5 minutes, birth weight 1.054 kg).

The patient was transferred to the intensive care unit (ICU) and extubated directly onto her noninvasive ventilator four hours later. Postnatally, respiratory function remained stable with room air SpO_2_ 96% and RR 24. She was transferred to the postnatal ward three days later, where she continued to express breast milk and had daily chest physiotherapy which consisted of insufflation via her volume ventilator with manual assisted coughing. As an abdominal thrust was inappropriate secondary to the abdominal wound, a ptussive squeeze was utilised. Mechanical inexsufflation was not required. She was discharged from hospital 10 days after delivery whilst her baby remained well in the neonatal ICU. Postoperative VC was measured at 0.21 to 0.23 L and was most likely pain limited. The neonate was transferred to a level 2 nursery at another hospital closer to the couple's home at a corrected age of 32 weeks. Three and a half months following discharge, the mother's VC had returned to 0.33 L, and review by a neonatologist found the infant to be developing normally. The child's weight (3.5 kg) and head circumference (35.5 cm) were increasing appropriately around the 10th percentile. The couple was happy to have the newborn monitored clinically for any signs of SMA and declined genetic testing.

### 2.2. 2nd Pregnancy

An unplanned second pregnancy was confirmed eight months later, with late presentation at 19 weeks gestation. Between pregnancies, there had been further deterioration in the patient's neuromuscular condition, with loss of left hand function removing control of her electric wheelchair. On first presentation, VC had fallen to 0.27 L (8% predicted). The antenatal care plan was duplicated between pregnancies. Respiratory function remained stable during the pregnancy (VC 0.24 to 0.32 L and SpO_2_ 94 to 98%), requiring only nocturnal nasal ventilation. These measurements are illustrated in Figure 1(b). Admission took place at 26 weeks for respiratory optimization, with VC at surgery being 0.28 L.

At 28 weeks and after steroid cover, an elective classical caesarean section with bilateral tubal sterilization (at the patient's request) was performed through the previous vertical abdominal midline incision. An elective awake mouthpiece-assisted fibreoptic intubation general anaesthetic was performed. A live, male infant was delivered (Apgars of 6 at 1 minute, 7 at 5 minutes, 8 at 10 minutes, birth weight 1 kg). Postoperatively, the patient was admitted to ICU, where she was extubated on to NIV three hours later and discharged to the obstetric ward two days later. During this time there was a minor increase in sputum which was coughed out effectively after volume ventilator insufflation and using manual assist techniques (ptussive squeeze). Oxygen saturations on room air remained ≥96% throughout her recovery period. Postpartum course was uneventful, and discharge took place on day seven.

## 3. Discussion

In normal pregnancies, total lung capacity is preserved with a small reduction in residual volume which generally stabilises or mildly enhances the VC. The major change in normal lung volumes is the reduction in functional residual capacity due to a reduction in chest wall compliance, starting early second trimester and worsening as the pregnancy progresses to a 35% to 40% decline [12]. In our patient it was felt that this could promote further microatelectasis and impede her ability to generate adequate tidal volumes.

For ongoing SMA pregnancies, antenatal care centres around maternal respiratory function, with NIV being shown to reduce respiratory compromise [10] as shown here. The benefits of training breath stacking to maximal insufflation have been described as important in preventing continued microatelectasis from chronically underventilated lungs and by maintaining the patients' chest wall range of motion to prevent further deterioration in VC [13]. Insufflation can be achieved effectively using a volume ventilator or a manual resuscitation bag and a one-way valve [13, 14]. Increase in insufflation capacity has been associated with the ability to generate more effective peak cough flows [13]. Manually assisted coughing [15, 16] and mechanical in-exsufflation [16] further enhance peak cough flows. In one study, the addition of insufflation capacity to unassisted inspiration in neuromuscular patients increased peak cough flows from 1.81 L/s to 3.37 L/s. Furthermore, adding manual abdominal assistance resulted in peak cough flows of 4.27 L/s, and mechanical in-exsufflation increased peak cough flows to 7.47 L/s [16]. In our patient where a growing uterus would put an increased load on her already small, stiff, and weak chest wall, it was paramount to try to maintain the patient's pulmonary compliance by breath stacking and to have practised techniques (including mechanical in-exsufflation) to promote early and successful extubation and effective secretion removal [17]. This was of special concern as her peak cough flows were well below the suggested threshold of 2.7 L/s [17] for effective airway clearance. However, mechanical inexsufflation was not utilised for this patient, as effective secretion clearance was achieved with insufflation and manual assisted coughing alone, and this method was preferred by the patient. In addition to the volume ventilator assisting with mouthpiece ventilation and breath stacking, it also ensures that a consistent tidal volume is delivered to the patient in the presence of changing respiratory compliance or resistance [18]. In contrast, with pressure preset devices (e.g., bi-level positive airway pressure) the patient cannot breath stack and tidal volumes delivered will vary based on changes in respiratory mechanics, likely to occur as pregnancy progresses. 

The importance of avoiding routine mask-oxygen prescription (without mechanical ventilation) in patients who are desaturating due to alveolar hypoventilation should be emphasized. In these scenarios supplemental oxygen alone can lead to reduced ventilatory drive, CO_2_ retention, and respiratory failure. It is for this reason that mechanical ventilation (usually NIV) is the primary treatment option for hypoventilation in neuromuscular disease [19, 20]. 

In order to assess the adequacy of ventilation, a measure of CO_2_ should be undertaken. ABGs provide the most accurate measure, but due to the difficulty in obtaining blood in this patient, routine measurements were not performed. As there were no signs of sustained deterioration on room air, obtaining such a sample was not pursued. However, in retrospect CO_2_ should have been monitored routinely. Other accurate measures include arterial capillary sampling from the ear lobe or finger pulp [21], arterialised venous sampling [22], and serial measures of serum bicarbonate. Careful interpretation is required as “normal” pregnancy baseline bicarbonate levels are lower at 15 to 20 mmol/L [12]. End-Tidal CO_2_ monitoring is a noninvasive measure which has been shown to correlate with PaCO_2_ in nonintubated patients breathing room air [23, 24]. However, the proximity of this relationship declines in the presence of ventilation-perfusion mismatching [24]. Transcutaneous CO_2_ is a less reliable measure and can over or under estimate PaCO_2_ results in adults [25].

During the outpatient period, SpO_2_ levels were measured weekly at one or two intervals. At this frequency of monitoring, a detection of respiratory deterioration may have been missed or delayed. In patients with access to domiciliary oximetry, acting on SpO_2_ <95% with mechanical in-exsufflation and increasing use of NIV can significantly decrease hospitalization rates for respiratory complications of neuromuscular disease [26]. Our patient presented with a baseline SpO_2_ of 96% and a range from 93 to 97%. At times when her SpO_2_ was abnormal (i.e., below 95%) a measure of CO_2_ would have assisted to clarify whether hypercapnia was present. Hypercapnia would likely indicate hypoventilation or secretion retention, both of which can be ameliorated by increasing NIV assistance and mechanical in-exsufflation [26].

Obstetric care during this pregnancy revolved around close regular obstetric surveillance, ongoing respiratory team review, admission for intensive observation, and a multidisciplinary decision regarding the optimum gestation for delivery. The latter decision is most likely to be open to discussion. In the absence of published literature on the precise effects of advancing gestation in a woman with Type 2 SMA weighing just 20 kg and with a VC of 11% predicted, it was difficult to be certain how much further incapacitation could be withstood before the mother's respiratory status was put at risk. Whilst it is feasible to provide continuous ventilatory support via daytime mouthpiece ventilation and nocturnal mask ventilation in patients with very low VC throughout pregnancy [11], our patient stated that she did not wish to use NIV support continuously. Management and delivery was therefore a balance of neonatal versus maternal mortality and morbidity, as is frequently the case in perinatal management. There is no literature on maternal outcome with such poor respiratory function and low pregnancy weight in SMA, though a limited amount of literature exists for other causes of respiratory compromise [11, 27, 28].

Anaesthetic review prior to labour and delivery is essential due to the respiratory compromise in SMA and the associated difficulties with both regional and general anaesthesia. Failed regional anaesthesia due to spinal column deformity is not uncommon in SMA, and the usefulness of subsequent awake fibreoptic technique has been reported [29]. General anaesthesia may be hazardous due to restriction in mouth opening, sensitivity to muscle relaxants, and hyperkalaemia postadministration of suxamethonium. Whilst caesarean section is the most common mode of delivery in SMA, women with more minor forms of this condition have adequate uterine function and can achieve a vaginal delivery. Previous case reports have documented a caesarean section for cephalopelvic disproportion at full dilatation after the spontaneous onset of labour, and an induction of labour at 37 weeks gestation with delivery by vacuum extraction, in women with Type 3 SMA with no respiratory compromise [30]. 

A literature review was performed by searching MEDLINE from 1950 to August 2008 using key words “spinal muscular atrophy”, “SMA,” and “pregnancy”. Seventeen case reports describing a total of 33 pregnancies were identified. The largest review was of 12 females with 17 infants [2]. Gestation at delivery of these women with more minor degrees of SMA ranged from 31–39 weeks. From our experience and the published literature, pregnancy in women with SMA can be successful despite potential complications. These women should obtain prepregnancy advice and antenatal care from a multidisciplinary team of healthcare professionals, in order to maximise outcomes for mother and baby and achieve informed consent.
